# AI-2/LuxS Quorum Sensing System Promotes Biofilm Formation of Lactobacillus rhamnosus GG and Enhances the Resistance to Enterotoxigenic Escherichia coli in Germ-Free Zebrafish

**DOI:** 10.1128/spectrum.00610-22

**Published:** 2022-06-14

**Authors:** Zhaoxi Deng, Kangwei Hou, Teresa G. Valencak, Xin M. Luo, Jianxin Liu, Haifeng Wang

**Affiliations:** a College of Animal Science, MOE Key Laboratory of Molecular Animal Nutrition, Zhejiang University, Hangzhou, P.R. China; b Department of Biomedical Sciences and Pathobiology, Virginia Polytechnic Institute and State University, Blacksburg, Virginia, USA; Griffith University

**Keywords:** *Lactobacillus rhamnosus* GG, *luxS* mutant, quorum sensing, biofilm, zebrafish, anti-inflammatory

## Abstract

The LuxS enzyme plays a key role in both quorum sensing (QS) and the regulation of bacterial growth. It catalyzes the production of autoinducer-2 (AI-2) signaling molecule, which is a component of the methyl cycle and methionine metabolism. This study aimed at investigating the differences between the Lactobacillus rhamnosus GG (LGG) wild-type strain (WT) and its *luxS* mutant (ΔluxS) during biofilm formation and when resisting to inflammation caused by Enterotoxigenic Escherichia coli (ETEC) in germ-free zebrafish. Our results suggest that in the absence of *luxS* when LGG was knocked out, biofilm formation, extracellular polysaccharide secretion and adhesion were all compromised. Addition of synthetic AI-2 indeed rescued, at least partially, the deficiencies observed in the mutant strain. The colonizing and immunomodulatory function in WT versus ΔluxS mutants were further studied in a germ-free zebrafish model. The concentration of AI-2 signaling molecules decreased sharply in zebrafish infected with the ΔluxS. At the same time, compared with the ΔluxS, the wild-type strain could colonize the germ-free zebrafish more effectively. Our transcriptome results suggest that genes involved in immunity, signal transduction, and cell adhesion were downregulated in zebrafish infected with ΔluxS and WT. In the WT, the immune system of germ-free zebrafish was activated more effectively through the MAPK and NF-κB pathway, and its ability to fight the infection against ETEC was increased. Together, our results demonstrate that the AI-2/LuxS system plays an important role in biofilm formation to improve LGG and alleviate inflammation caused by ETEC in germ-free zebrafish.

**IMPORTANCE** Lactobacillus rhamnosus GG is a widely used probiotic to improve host intestinal health, promote growth, reduce diarrhea, and modulate immunity. In recent years, the bacterial quorum sensing system has attracted much attention; however, there has not been much research on the effect of the LuxS/AI-2 quorum sensing system of *Lactobacillus* on bacteriostasis, microbial ecology balance, and immune regulation in intestine. In this study, we used germ-free zebrafish as an animal model to compare the differences between wild-type and *luxS* mutant strains. We showed how AI-2/LuxS QS affects the release of AI-2 and how QS regulates the colonization, EPS synthesis and biofilm formation of LGG. This study provides an idea for the targeted regulation of animal intestinal health with probiotics by controlling bacteria quorum sensing system.

## INTRODUCTION

The gastrointestinal, respiratory tract and genital tract are colonized with a myriad of bacteria, archaea, fungi, and viruses, which are summarized collectively as the “microbiota” (1). The intestine is home to a large and complex sex of microbiota. The microbiota play an important role in regulating the host's intestinal health (2). The main ecological niche for Lactic acid bacteria is located in the gastrointestinal tract, and it is adapted to various conditions with changes in their metabolism accordingly (3). Successful colonization in gastrointestinal tract is a key step for Lactic acid bacteria to exert a sufficient host-interaction to confer health benefits (4). Lactobacillus rhamnosus GG (LGG) is one of the most widely used probiotic strains having beneficial effects on intestinal function, including stimulating the development of mucosal immunity, maintaining and improving the intestinal barrier function, and regulating overall immunity in gastrointestinal tract (5–7).

Quorum sensing (QS) system is a means for bacteria to exchange information using signal molecules (8). It must be emphasized that QS plays an important role in regulating the formation of biofilms (9). Generally, bacterial cells produce a variety of signaling molecules (such as AI-2), the autoinducers, whose extracellular concentration increases with increasing cell density (10). Eventually, the target gene is activated to show certain physiological functions that a single cell cannot perform independently. A variety of Gram-positive and Gram-negative bacteria produce AI-2 through a common biosynthetic pathway, and it has been suggested that AI-2 functions in intra- and inter-species communication (11). The canonical biosynthetic pathway of AI-2 is part of the activated methyl cycle. AI-2 uses methionine as starting material and is produced through four enzymatic steps. Methionine is catalyzed by S-adenosylmethionine synthase (MetK) to produce S-adenosylmethionine (SAM). SAM acts as a methyl donor to produce S-adenosylhomocysteine (SAH) that is hydrolyzed by S-adenosylhomocysteine nucleosidase (Pfs) to S-riboadenosylhomocysteine (SRH) and adenine. LuxS, a protein encoded by the *luxS* gene, catalyzes SRH cleavage to form homocysteine and 4,5-dihydroxy 2,3-pentanedione (DPD). The former further generates methionine and enters the methyl cycle, while DPD is rearranged to produce AI-2 (12).

Bacteria exist in nature in the form of free-floating plankton or sessile colonies of microbes forming biofilms (13). Biofilm is an organized microbial aggregate that is embedded within a matrix of bacterial polysaccharides (14). The general process of biofilm formation is as follows: planktonic bacteria initially attach to the surface, and the exopolysaccharides (EPS) wrap the bacteria by forming microcolonies before, aggregating to form a mature biofilm. Finally, the biofilm is dispersed (15). At present, it has been confirmed that the LuxS/AI-2 QS system regulates the formation of biofilms. For *Lactobacillus*, the QS system primarily affects the growth and dispersal phases of biofilm formation. Although not all the functions of EPS in bacterial cells have yet been elucidated, it is established that they protect from harmful environmental influences and facilitate colonization by forming special structures stabilizing the biofilm (16). One study compared survival and persistence of a genuine *luxS* mutant and a *luxS* mutant containing an unknown suppressor mutation of LGG in the wild- type of the gastrointestinal tract in mice. Thus, EPS might play an important role in biofilm formation (17). After the deletion of the *luxS* gene in LGG, the ability of biofilm formation was decreased, and exogenous supplementation of DPD (AI-2 precursor) or the supernatant of the wild-type could complement biofilm formation of the mutant (18). However, in Lactobacillus reuteri 100-23C, biofilm formation was significantly enhanced after the deletion of *luxS* gene compared with the wild type, while exogenous addition of DPD did not attenuate biofilm formation (19). It is an open question if the regulation of the LuxS/AI-2 QS system on biofilm formation in different bacteria works in the same manner.

Zebrafish (Danio rerio) have become a popular animal model in recent years. They can replace other classical animal models due to their high fecundity, small size, and simple mode of operation for body and real-time imaging (20). Intestinal villi from zebrafish have goblet cells that secrete mucus, which can accurately simulate bacterial adhesion and colonization. Therefore, zebrafish are often used as a model for studying host-bacteria interactions (21, 22). Since the zebrafish have 85% gene homology with humans, it also is a suitable model system for inflammatory bowel disease (23). Previous studies have shown a positive effect of the β-glucan-producing Pediococcus parvulus 2.6 on the colonization of the zebrafish gut, as well as on the competition of the bacterium with the pathogen Vibrio anguillarum in such an environment (24). Lactobacillus sakei MN1 efficiently colonizes the zebrafish gut and inhibits the limination of Vibrio anguillarum NB10 (25). Therefore, *Lactobacillus* may colonize the intestinal tract of zebrafish and exhibit positive health effects (24, 26). At present, most studies were done *in vitro* by comparing wild-type and *luxS* mutants. In our study, we tested how AI-2/LuxS QS might affect the release of AI-2 and how colonization is regulated along with EPS synthesis and biofilm formation of LGG. We used germ-free zebrafish as an animal model to explore the differences in the regulation of inflammation between wild-type and △luxS *in vivo*. We aimed at revealing the relationship between biofilm formation and intestinal immunity, and at using the *luxS*/AI-2 QS system to fight inflammation.

## RESULTS

### Characterization of a novel luxS mutant shows reduced AI-2 activity.

**FIG 1:**
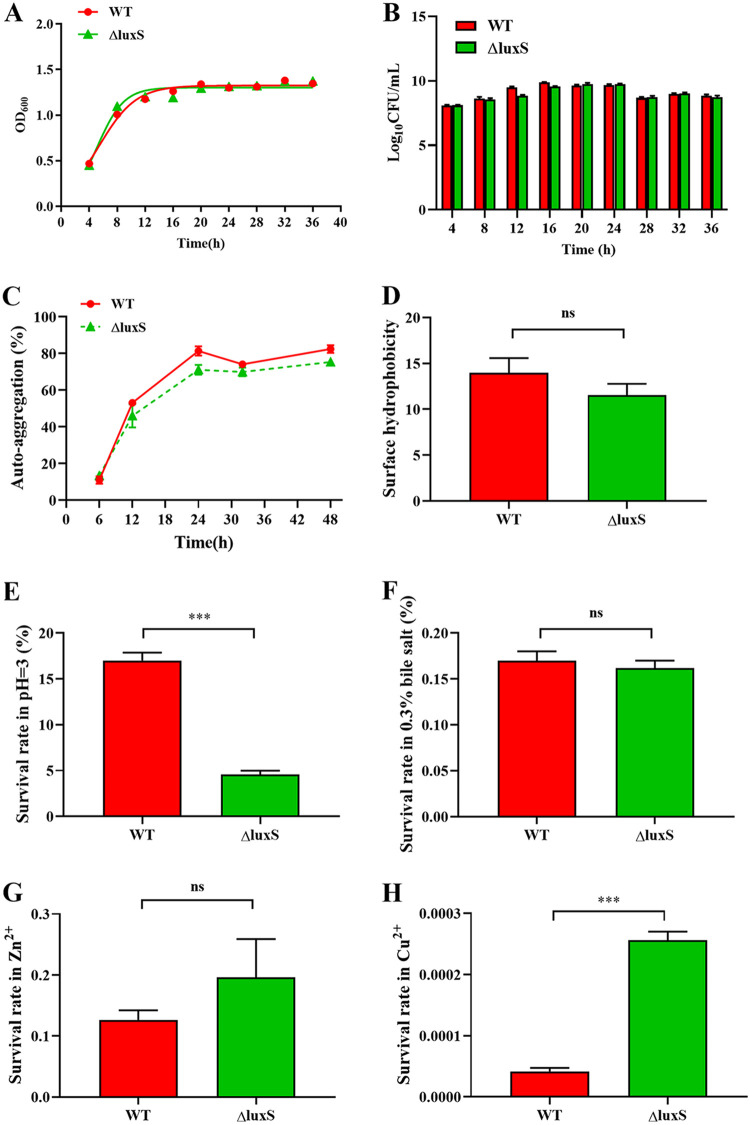
Physiological characteristics and stress resistance in WT and ΔluxS strain. (A–B) WT and ΔluxS were counted after being cultured for 4, 8, 12, 16, 20, 24, 28, 32 and 36 h in MRS. (C) Autoaggregation. (D) Surface hydrophobicity. (E-H) Survival rates in hydrochloric acid, bile salts (0.3%), copper ions and zinc ions (100 mg/L), *n* = 6. WT, wild type; ΔluxS, *luxS* mutant; *P < *0.001 (***).

In order to delete *luxS*, we applied a vancomycin-based counter-selection system (pVPL3002), and finally obtained the deleted strain through homologous recombination. Our results suggest that there were no significant differences in growth characteristics (Fig. 1A and B), auto-aggregation (Fig. 1C), surface hydrophobicity (Fig. 1D), stress resistance to bile salts (Fig. 1F) and zinc ions (Fig. 1G) between the WT and ΔluxS strain (Fig. 1). Compared with the wild type, the ΔluxS strain was significantly less tolerance to acids (pH 3.0) (Fig. 1E), but significantly more tolerance toward copper ions (Fig. 1H).

**FIG 2:**
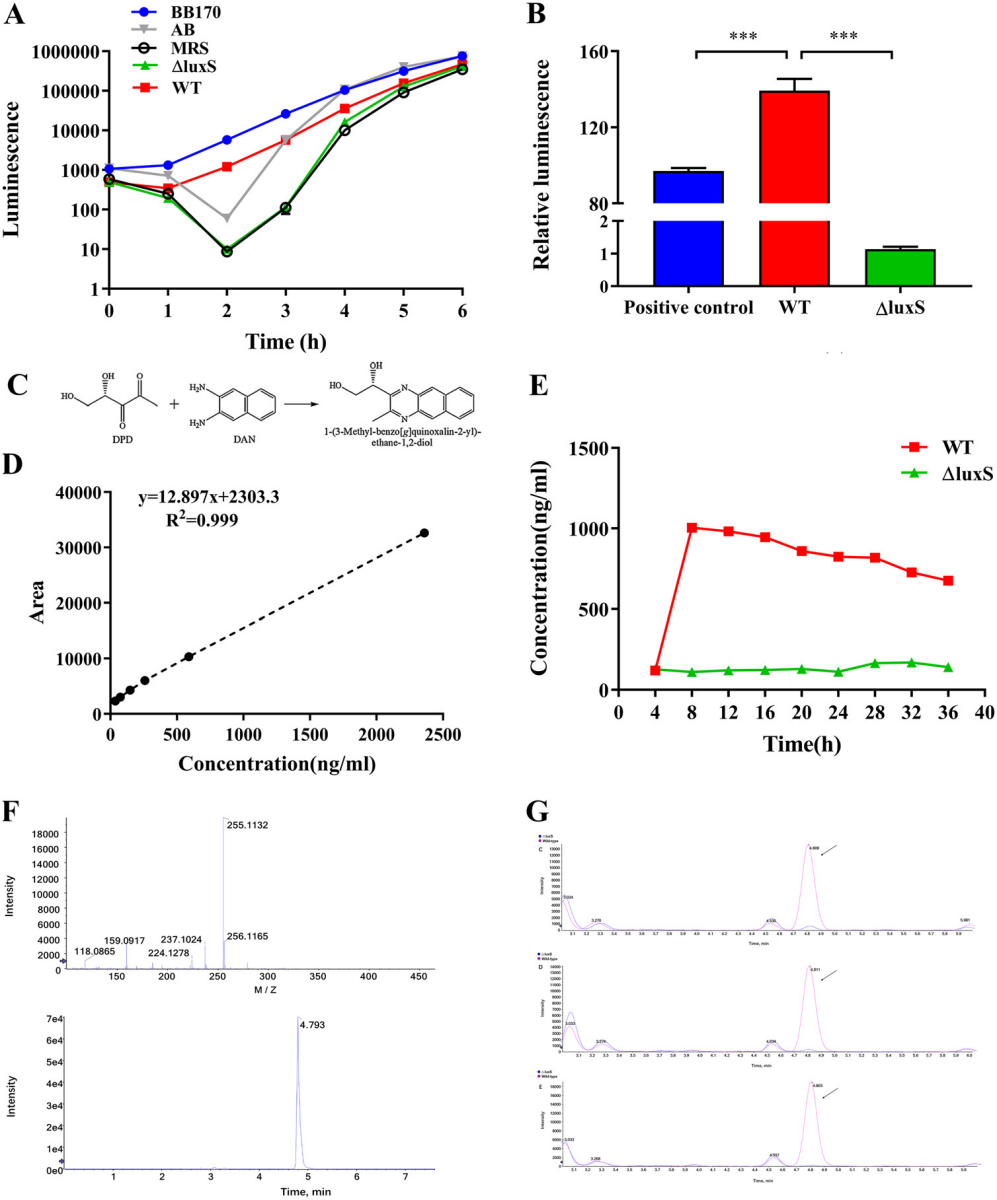
AI-2 activity of LGG wild-type or ΔluxS. (A) Detection of AI-2 activity in the supernatant of the wild-type strain and ΔluxS mutant after inducing V. harveyi BB170 to emit light. The supernatant of V. harveyi BB170, AB liquid medium, and MRS liquid medium were used as positive, negative and medium controls, respectively, *n* = 6. (B) Relative luminescence compared to the negative control at 24 h, *n* = 6. *P < *0.001 (***). (C) The process of 4,5-dihydroxy 2,3-pentanedione (DPD) derivatization. It was an atypical condensation reaction with the loss of two water molecules. (D) Standard curve of AI-2 detected by HPLC-FLD. (E) Quantitative detection of AI-2 in the supernatant of LGG strains from different time points by HPLC-FLD. (F) MS spectra of the derived product of DPD. (G) Representative chromatograms of LGG supernatant (8h, 16h, 24h). WT, wild type; ΔluxS, *luxS* mutant; red, wild type; blue, ΔluxS strain.

As Vibrio harveyi BB170 can be activated as reporter strain by AI-2 to excite luminescence, luminescence intensity can be used to evaluate the amount of AI-2. The qualitative detection of AI-2 by the V. harveyi BB170 reporter strain suggested that after reaching the lowest point in each group, luminescence increased with time. The ΔluxS strain, media control and negative control had the lowest luminescence at 2 h, whereas the LGG wild-type strain and positive control had the lowest luminescence at 1 h (Fig. 2A). The relative amount of the AI-2 signaling molecule was measured by luminescence intensity before and after induction. We found that the relative luminescence of the LGG wild-type strain supernatant was significantly higher than that of the positive control, with the Δ*luxS* strain hardly producing any AI-2 (Fig. 2B). During HPLC analysis, the derivation of 4,5-dihydroxy 2,3-pentanedione (DPD) was carried out by reaction with 2,3-Diaminonaphthalene (DAN) to produce 1-(3-methyl-bezo[g]quinoxalin-2-yl)-ethane-1,2-diol (Fig. 2C). The standard curve was set up based on the peak area with different loading concentration (Fig. 2D). The quantitative detection of AI-2 by HPLC-FLD showed that the concentration of AI-2 released by LGG was highest at 8 h, then decreased slowly, while the ΔluxS strain hardly produced any AI-2 (Fig. 2E). Further verification of AI-2 by LC-MS/MS suggested that the formula of the protonated molecule ([M+H]^+^) of the derived product was C_15_H_15_O_2_N_2_ and the mass of ion (*m/z*) was 255.11 (Fig. 2F). Under the same conditions, the peak time of the standard was about 4.8 min. Supernatant collected from LGG cultured at 8 h, 16 h, and 24 h showed a peak at the same time point, while no peak was present for the ΔluxS strain at this time (Fig. 2G).

### Cell adhesion and biofilm formation in the LGG wild-type and ΔluxS strain.

**FIG 3:**
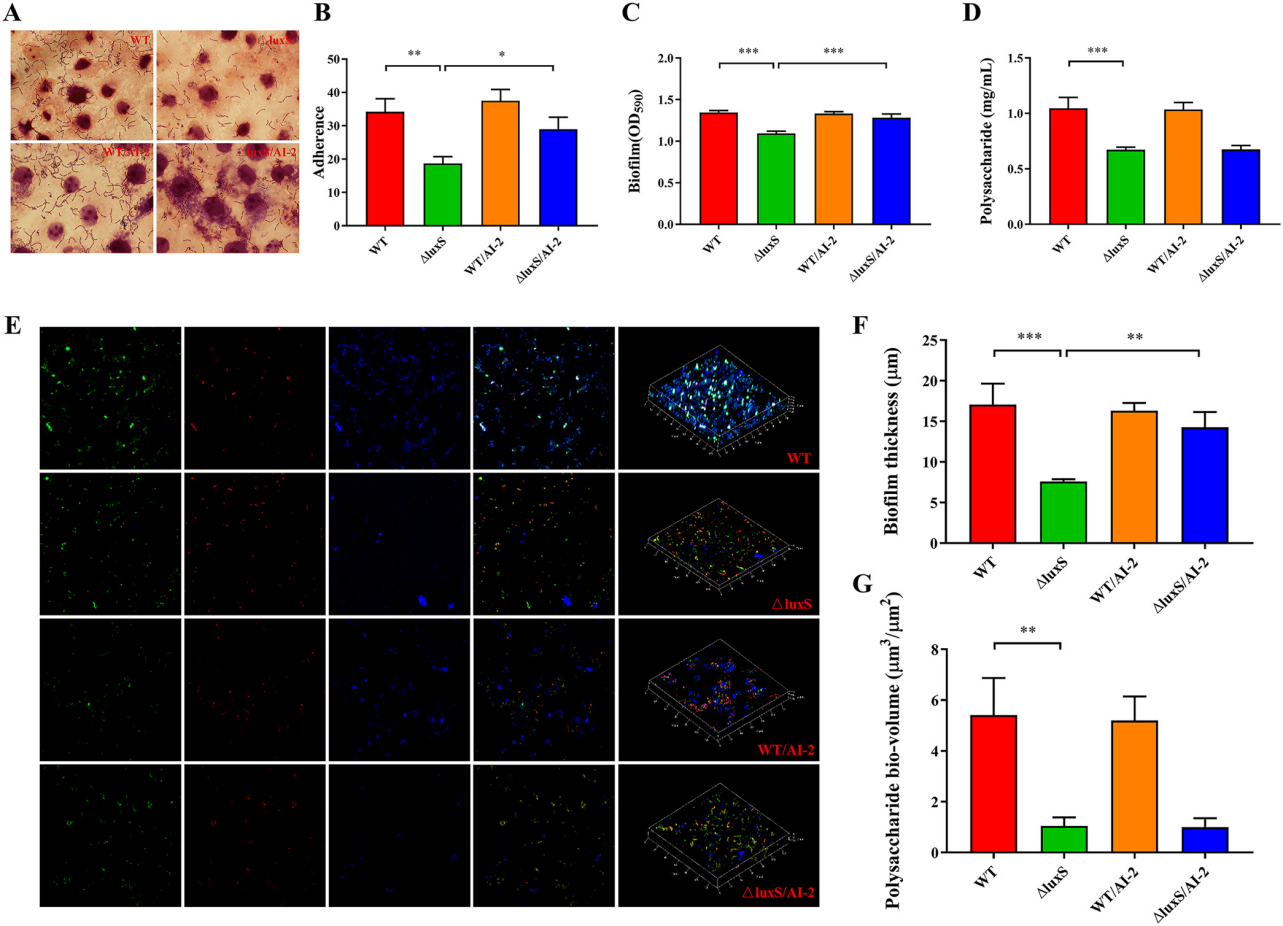
Adherence capacity, biofilm formation and exopolysaccharide (EPS) production of the LGG wild type versus △luxS in the presence or absence of exogenous, synthesized AI-2 (1 μM). (A–B) Adherence of LGG strains to IPEC-J2 cells. Five fields of vision were randomly selected for each slide under oil microscope to calculate the number of bacteria adhered to the surface of the visible cells, *n* = 6. (C) Biofilm determined by crystal violet staining, *n* = 6. (D) EPS produced in the biofilm matrix, *n* = 6. (E-G) Confocal laser scanning microscopy (CLSM) staining of living cells, dead cells, and EPS by SYTO9 (green), PI (red) and calcofluor white (blue), respectively, *n* = 3. AI-2, autoinducer-2; WT, wild type; ΔluxS, *luxS* mutant; WT/AI-2, wild-type strain supplemented with AI-2; ΔluxS/AI-2, ΔluxS strain supplemented with AI-2. *P < *0.05 (*), *P < *0.01 (**) and *P < *0.001 (***).

The ability to adhere to IPEC-J2 cells was compared between wild-type and ΔluxS strain with or without supplementation of AI-2 as a rescue. The concentration of the wild-type strain in the supernatant of AI-2 used was measured by HPLC (1 μM). Compared with the wild type, the ΔluxS strain had significantly lower adhesion to IPEC-J2. While supplementation with AI-2 significantly improved the adhesion of the ΔluxS strain to IPEC-J2 compared with the ΔluxS strain alone, it did not affect adhesion of the wild-type strain to IPEC-J2 (Fig. 3A and B).

Crystal violet staining in the 96-well microtiter plate was used to analyze the differences in biofilm formation between wild-type and ΔluxS strain with or without supplementation with AI-2. The ΔluxS strain had significantly weaker biofilm formation than the wild-type strain after being cultured for 24 h. Supplementation with AI-2 rescued biofilm formation of the ΔluxS strain, however, the supplementation had no effect on biofilm formation in the wild-type strain (Fig. 3C). The polysaccharide content in the biofilm matrix detected by the phenol-sulfuric acid method was significantly lower in the ΔluxS strain than in the wild type, while AI-2 supplementation did not affect either (Fig. 3D). We further visualized biofilm formation through CLSM and characterized thickness and polysaccharide bio-volume (Fig. 3E). The wild-type strain developed a thicker mature biofilm and produced more polysaccharides than the ΔluxS strain. AI-2 increased the thickness of ΔluxS biofilms; however, it did not increase the release of polysaccharides (Fig. 3F and G). In summary, our results confirm that the expression of *luxS* regulates adherence and biofilm formation of LGG.

### Colonization of LGG strains in germ-free zebrafish.

**FIG 4:**
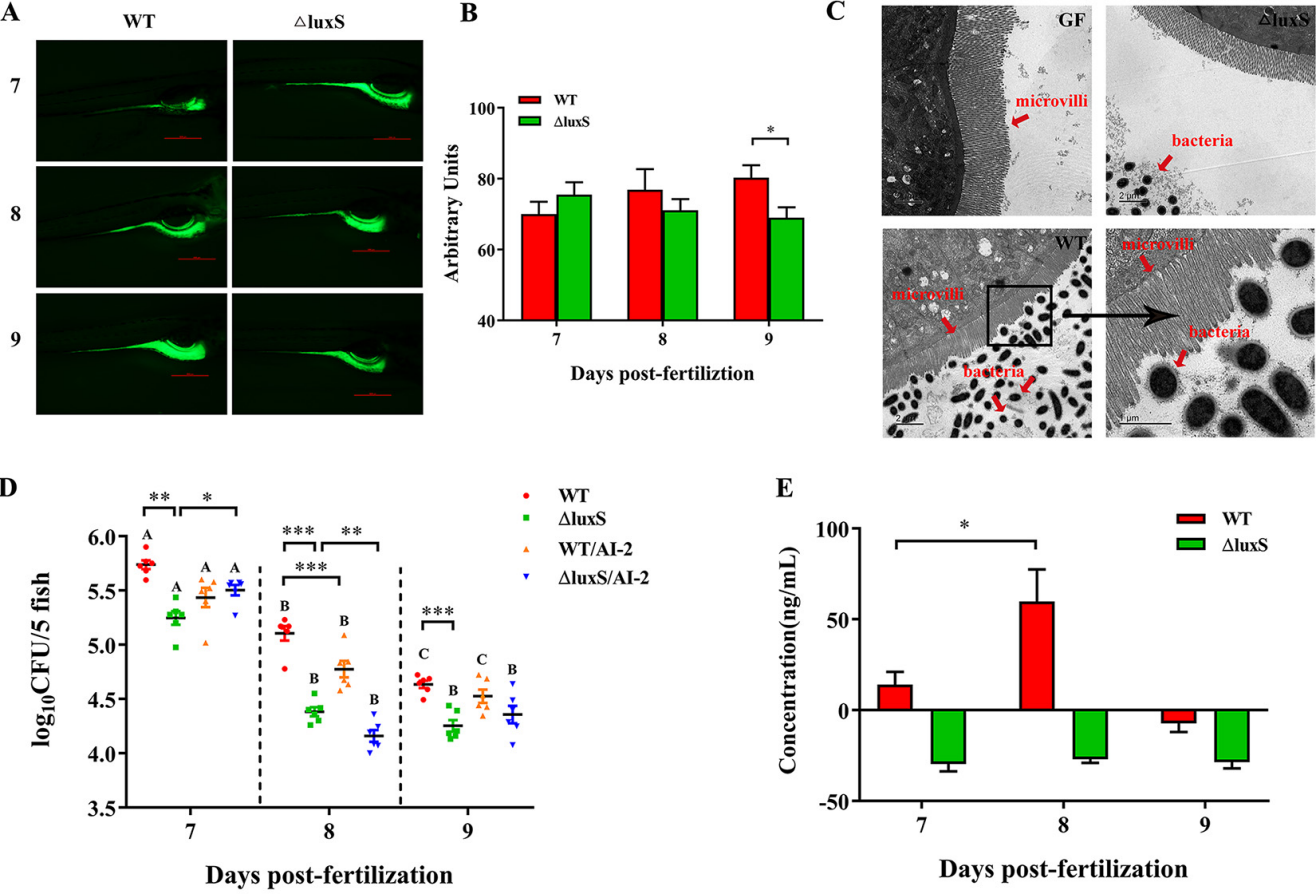
Colonization of germ-free zebrafish larvae by LGG wild-type and ΔluxS strain. (A–B) Analysis of LGG colonization in zebrafish gut on 7, 8 and 9 dpf by fluorescence microscopy, *n* = 9-12. Scale bar: 500 px. (C) Analysis of LGG colonization in zebrafish gut on 6 dpf by transmission electron microscopy. The figure shows germ-free zebrafish (GF), as well as germ-free zebrafish immersed in WT and ΔluxS. Red arrows point to microvilli and bacteria, respectively. Scale bar: 2 μm or 1 μm. (D) Colony-forming unit (CFU) counts of zebrafish larvae in the presence or absence of exogenous, synthesized AI-2 (1 μM), *n* = 6. (E) Determination of AI-2 content in zebrafish on 7, 8 and 9 dpf by HPLC-FLD, *n* = 3. AI-2, autoinducer-2; WT, wild type; ΔluxS, *luxS* mutant; WT/AI-2, wild-type strain supplemented with AI-2; ΔluxS/AI-2, ΔluxS strain supplemented with AI-2. *P < *0.05 (*), *P < *0.01 (**) and *P < *0.001 (***). A negative value in the figure indicates that its concentration is too low and did not meet the detection baseline.

We produced germ-free zebrafish larvae by sterilizing fertilized eggs with both antibiotic and chemical treatments. A fluorescent probe for quantitative detection of the transcription levels of toll-like receptor molecules (TLR1, TLR2, TLR3, TLR4b and TLR5b) was used to monitor the activation of the innate immune system. The expression levels of TLR molecules were very low in germ-free (GF) zebrafish, and higher in conventionally raised (CR) zebrafish, indicating that the innate immune system was activated by the microbe-associated molecular patterns (MAMPs) of microorganisms. After germ-free zebrafish were inoculated with wild-type or ΔluxS LGG strains, TLR1, TLR2, TLR3 and TLR5 were significantly upregulated (Fig. S1). Both fluorescence microscopy and bacteria count test confirmed that wild-type and ΔluxS LGG colonized the gut until at least 9 dpf (Fig. 4A, B to D). However, the capacity of the wild-type strain to colonize the gut was significantly higher than that of ΔluxS. Supplementation with AI-2 did not have an effect on the colonization of the WT strain (Fig. 4D). Moreover, transmission electron microscopy showed that there were no bacteria in GF zebrafish intestines. In contrast, a large amount of the wild-type strain existed in the gut and interacted with the intestinal villi, while only a few ΔluxS bacteria were found in the intestinal lumen and detach from the villi (Fig. 4C). The quantitative detection of AI-2 in zebrafish by HPLC-FLD indicated that the concentration of AI-2 was the highest in the wild-type group at 8 dpf. AI-2 was not detected in zebrafish treated with ΔluxS (Fig. 4E). These results suggest that knockouting *luxS*, the ability of LGG to colonize the gut of GF zebrafish is significantly reduced.

### Gene expression in zebrafish larvae.

**FIG 5:**
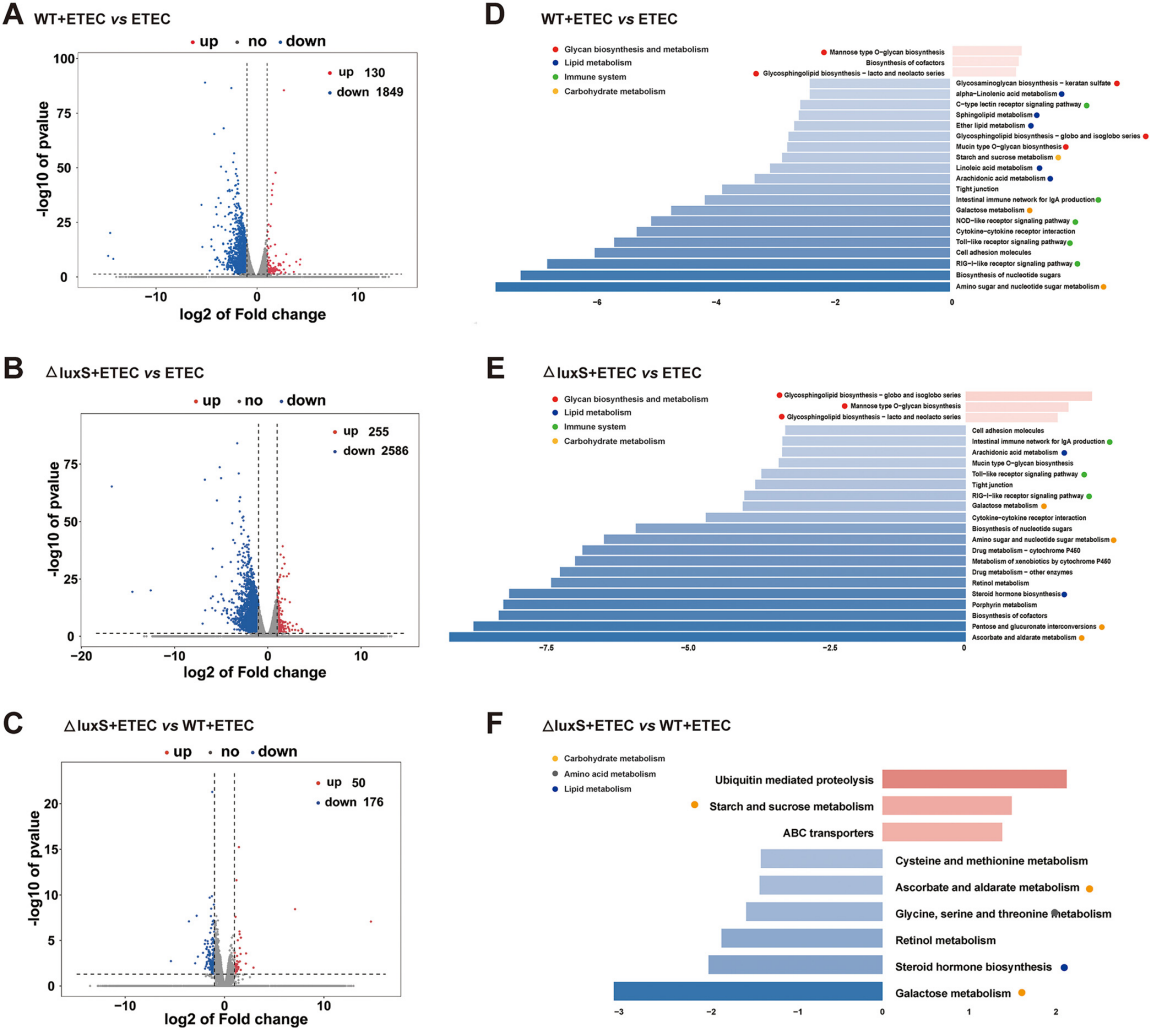
Transcriptomic analysis of zebrafish larvae. (A–C) Volcano plots of DEGs. Log_2_ (Fold change) ≥1 was set as the threshold. (D–F) Pathway classification based on KEGG enrichment analysis of DEGs. We enriched the differential genes according to Log_2_ (Fold change) ≥1, upregulated genes *P < *0.1, and downregulated genes *P < *0.05 for KEGG functional enrichment. We selected the top 20 pathways for presentation. Red: Glycan biosynthesis and metabolism. Blue: lipid metabolism. Green: immune system. Yellow: carbohydrate metabolism. Gray: amino acid metabolism. ETEC, WT+ETEC and ΔluxS+ETEC, zebrafish larvae in germ-free were exposed to none or to wild-type or ΔluxS LGG at a concentration of 10^8^ CFU/mL for 24 h on 5 dpf, respectively, before being exposed to ETEC at a concentration of 10^8^ CFU/mL for 24 h on 6 dpf.

In order to explore the differences between the WT and ΔluxS strain, we used mRNA-seq to perform high-throughput gene expression analysis. Zebrafish larvae in germ-free exposed to none or to wild-type or ΔluxS LGG at a concentration of 10^8^ CFU/mL for 24 h on 5 dpf were designated as the GF, WT and ΔluxS group, respectively. These zebrafish larvae treated differently were further exposed to ETEC at a concentration of 10^8^ CFU/mL for 24 h on 6 dpf and considered as the ETEC, WT+ETEC and ΔluxS+ETEC groups, respectively. A total of 1979 genes were differentially expressed between the WT+ETEC and the ETEC group. Of these, 130 genes were upregulated and 1849 genes were downregulated in the WT+ETEC group compared to the ETEC group (Fig. 5A). A total of 2841 genes were differentially expressed between the ΔluxS+ETEC and the ETEC group. Among them, 255 genes were upregulated, and 2586 genes were downregulated in the ΔluxS+ETEC group (Fig. 5B). A total of 226 genes were differentially expressed between the ΔluxS+ETEC and WT+ETEC group. Compared to the WT+ETEC group, 50 genes were upregulated and 176 genes were downregulated in the ΔluxS+ETEC group (Fig. 5C). The DEGs between WT+ETEC and ETEC, and those between ΔluxS+ETEC and ETEC, are part of the immune system, of lipid and carbohydrate metabolism, and the genes were all downregulated (Fig. 5D and E). The DEGs between WT+ETEC and ΔluxS+ETEC, on the other hand, were involved in carbohydrate, amino acid and lipid metabolism (Fig. 5F).

### Modulation of inflammatory responses by wild-type and ΔluxS LGG strain.

**FIG 6:**
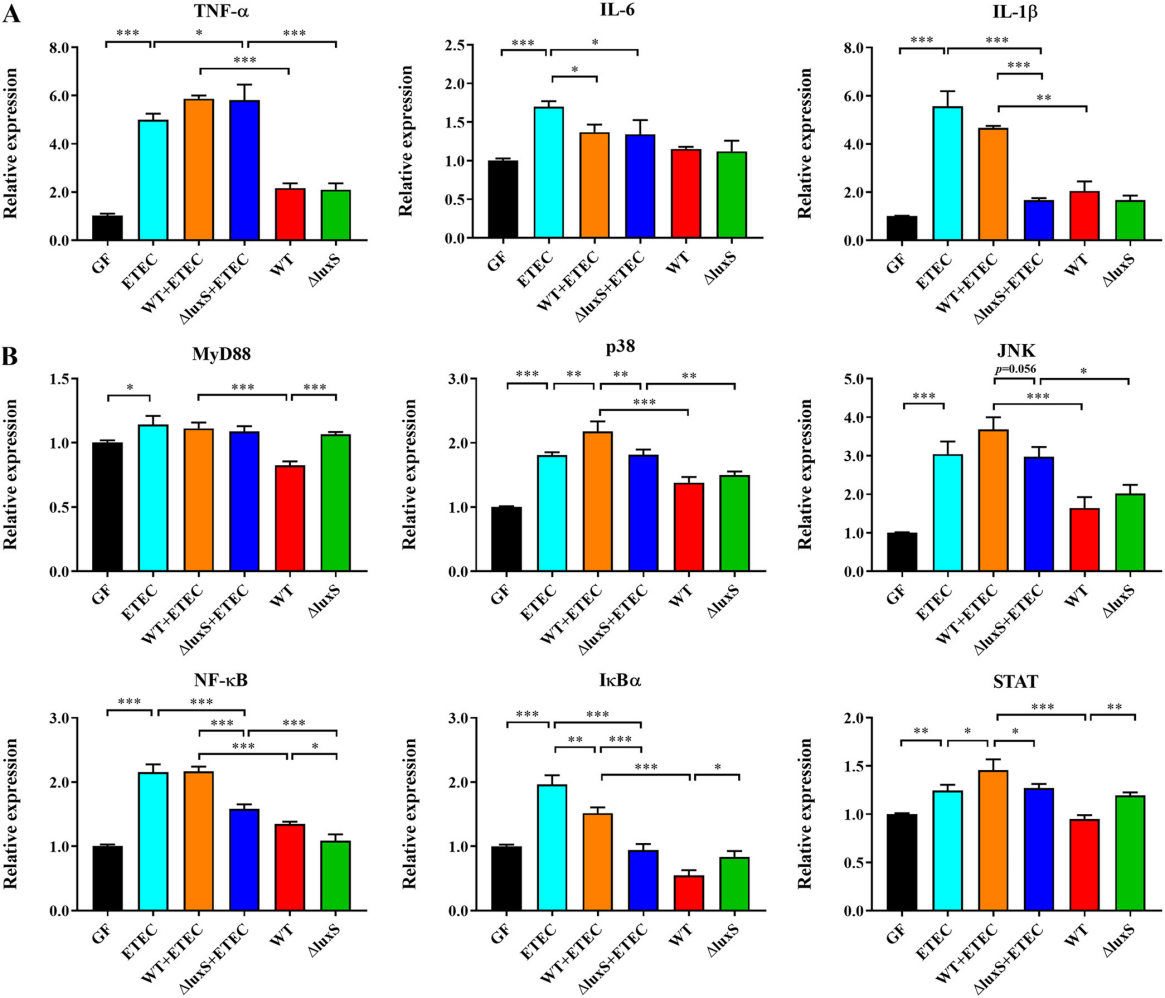
Characterization of inflammatory response in germ-free zebrafish larvae expose to ETEC. (A) mRNA expression of tumor necrosis factor-α (TNF-α), interleukin-6 (IL-6) and interleukin-1β (IL-1β). (B) mRNA expression of genes involved in mitogen-activated protein kinases (MAPK), nuclear transcription factor-kappa B (NF-κB) and Janus kinase/signal transducer and activator of transcription (JAK/STAT) signaling pathway, *n* = 6. GF, WT and ΔluxS, zebrafish larvae in germ-free were exposed to none or to wild-type or ΔluxS LGG at a concentration of 10^8^ CFU/mL for 24 h on 5 dpf, respectively. ETEC, WT+ETEC and ΔluxS+ETEC, zebrafish larvae in germ-free were exposed to none or to wild-type or ΔluxS LGG at a concentration of 10^8^ CFU/mL for 24 h on 5 dpf, respectively, before being exposed to ETEC at a concentration of 10^8^ CFU/mL for 24 h on 6 dpf. *P < *0.05 (*), *P < *0.01 (**) and *P < *0.001 (***).

Enterotoxigenic Escherichia coli (ETEC) is the most common pathogen causing intestinal infections by colonizing the intestine and synthesizing and secreting enterotoxin (27). The degree of inflammation is dependent on the colonization number of ETEC (28). To find out whether or not wild-type LGG or ΔluxS strain might have anti-inflammatory effects, we used RT-qPCR to examine the expression levels of genes in the zebrafish larvae (7dpf) relating to inflammation. Compared to the control group (GF), an ETEC infection significantly increased the expression levels of pro-inflammatory cytokines TNF-α, IL-6 and IL-1β of zebrafish larvae (Fig. 6A). Compared with the ETEC group, supplementation of wild-type and ΔluxS strain well in advance significantly downregulated the levels of IL-6 (Fig. 6A). We quantified the genes involved in MAPK and NF-κB signaling pathway to better understand the anti-inflammatory mechanism of LGG. All genes relating to MAPK and NF-κB pathway were significantly upregulated in zebrafish larvae in the ETEC group compared to the control group. Compared with the ETEC group, prior supplementation with ΔluxS strain significantly downregulated the NF-κB and IκBα levels (Fig. 6B).

## DISCUSSION

A large number of studies have confirmed a significant role of the AI-2/LuxS system in Gram-positive and Gram-negative bacteria sensing environmental changes by AI-2 signaling molecules. It drives physiological and biochemical functions of bacteria, including biofilm formation, bioluminescence, and the secretion of virulence factors (29, 30). Previous studies performed in Lactobacillus rhamnosus GG showed that *luxS* is essential for the synthesis of AI-2 (31, 32). Our study confirmed that *luxS* is essential for the synthesis of AI-2 in LGG, which is consistent with earlier findings in LGG. However, we further found that the lack of *luxS* did not completely prevent synthesis of AI-2. The ΔluxS strain could still produce very small amounts of AI-2. Although *luxS* might represent the main pathway for synthesizing AI-2, other pathways may also synthesize AI-2. It was reported that AI-2 can be obtained in the absence of *luxS* from spontaneous conversion of ribose-5-P (33). Another semi-biosynthetic pathway of AI-2 was identified from hyperthermophiles. With no *luxS* but only Pfs, hyperthermophiles cleave SAH into adenosine and homocysteine, and adenosine was isomerized to ribose-5-P. It is further converted into DPD and AI-2 by induction of heat (34). Both the wild-type and ΔluxS strain show no significant difference in growth characteristics, self-aggregation, surface hydrophobicity, and resistance to bile salts and zinc ions. Rather, the lack of *luxS* did not significantly affect the physiological characteristics of LGG. Compared with the wild type, the copper ion resistance of the ΔluxS strain was significantly increased. Acid tolerance was significantly reduced in the ΔluxS strain compared with the wild type, indicating that the wild type could adapt to the acidic environment more effectively.

The concentration of QS signaling molecules in biofilms can be 1,000 times higher than in environments where planktonic bacteria reside (35). It was reported that *luxS* of LGG might play a central, metabolic role in biofilm formation. Deletion of the *luxS* in LGG has reduced the ability to form biofilms. Our study showed that biofilm formation and adhesion were all weaker in the ΔluxS strain compared with the wild type. On this basis, we further explored its functions by addition synthetic AI-2 signaling molecules. Our results show that the adding synthetic AI-2 restored biofilm formation and adhesion of the ΔluxS strain. Consistently, deletion of the *luxS* of LGG reduced the ability to form biofilms. Exogenous addition of DPD or wild-type strains can partially replenish biofilm formation but cannot establish the original state (31). *pfs* encodes Pfs, which functions upstream of LuxS in the biosynthesis of AI-2. The Riemerella anatipestifer (RA) genome has *pfs* but no *luxS* homologue and does not produce AI-2. After additional administration of AI-2 signaling molecule to the medium, the expression levels of 13 genes relating to biofilm formation were significantly decreased, and the extent of biofilm formation was also significantly reduced. This suggests that although RA cannot generate AI-2 signaling molecules, it is influenced by AI-2 signaling molecules (36). Biofilms are defined as aggregates of microorganisms embedded in polysaccharides secreted by them. EPS-mediated changes support cellular recognition and further stimulate adhesion and aggregation (37, 38). Therefore, we examined the effect of *luxS* deletion on the production of exopolysaccharides by LGG. One recent study showed that the amount of EPS was significantly improved with the addition of different concentrations of AI-2 in Lactobacillus plantarum (39). In our study, the decrease expression of polysaccharide biosynthesis genes in the ΔluxS strain is consistent with the lower amounts of EPS determined by the phenol-sulfuric acid method and CLSM. However, addition of synthetic AI-2 could either fully restore the EPS secretion of the ΔluxS strain, nor could it promote the secretion of the wild type. It is possible that EPS is a major component of biofilm, but protein and extracellular DNA are also important components (14). Deletion of *luxS* affected transcription of a large number of genes, some of which may be involved in the expression of various biofilm components. Therefore, up- or downregulation of some of these genes might also affect EPS secretion. Altogether, we suggest that AI-2/LuxS has an important role in biofilm formation.

Previous studies showed that quorum sensing signaling molecules participate in maintaining barrier function, controlling inflammatory processes and increasing resistance to pathogens through interaction with receptors (40). Therefore, an in-depth understanding of the AI-2/LuxS QS system will turn out helpful for identifying the mechanism behind the LGG probiotic effect. We selected germ-free zebrafish as the model to further explore whether AI-2/LuxS will have an immunomodulatory function on the host through influencing pathogen colonization. The adhesion process of *Lactobacillus* is usually influenced by various internal and external environmental factors, including pH, bacterial growth dynamics, auto-aggregation, and surface hydrophobicity (41). We observed that the wild-type strain had a longer retention time in the gut, and the amount of AI-2 in the body decreased due to excretion of the bacteria. The wild-type strain had higher acid tolerance than the ΔluxS strain, which may contribute to the better adhesion and higher number of wild-type LGG in the zebrafish gut. Studies have confirmed that the front stomach of mice is the main habitat of Lactobacillus reuteri. Host acid secretion exerts a tremendous selective pressure on the Lactobacillus reuteri population, which makes acid resistance a key factor for successful colonization (42). Energy consumption often plays a vital role in the development of inflammation. Starch and sucrose metabolism, amino sugar metabolism, and galactose metabolism were all involved as pathways relating to carbohydrates in our study. The results from the transcriptome confirmed that LGG could promote host resistance to ETEC invasion by downregulating the innate immune system pathways such as RIG-I-like receptors, NOD-like receptors and toll-like receptor signaling. Our transcriptome results also suggested that glycine, serine and threonine metabolic pathways were all downregulated in the ΔluxS+ETEC group compared to the WT+ETEC group. Threonine is one of the most abundant essential amino acid in immunoglobulins, which is also a component of mucus glycoprotein acting as most extensive important barrier against virus invasion (43). Glutathione is composed of glycine, glutamate, and cysteine, and plays an important role in nutrient metabolism and intestinal immunity (44–46). These results indicate that amino acids were redistributed to fight the immune response and they play a key role in improving the integrity and function of the intestinal barrier.

**FIG 7:**
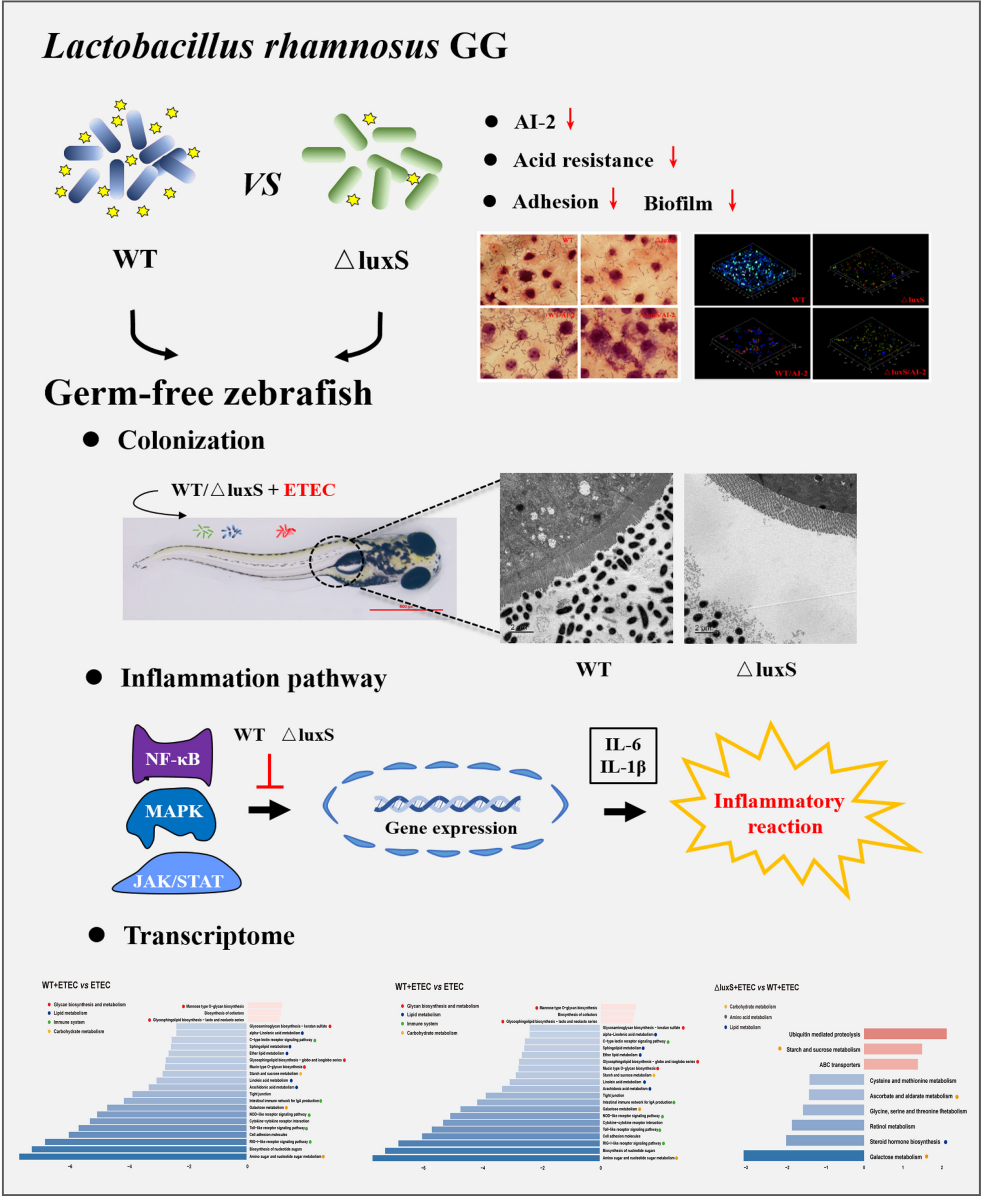
Abstract graphic. Our study compared the difference between the wild-type strain (WT) of Lactobacillus rhamnosus GG (LGG) and its *luxS* mutant strain (ΔluxS). In addition, the colonization and immunomodulatory effects of WT and ΔluxS were evaluated in the germ-free zebrafish model.

The colonization of germ-free zebrafish with a single bacterial species provides an opportunity to study the bacteria-host immune response of. Due to the confounding effect of local symbionts in conventionally raised fish, the response of germ-free and conventionally raised zebrafish to infection was significantly different. Our results show that after stimulation with WT and ΔluxS, the expression of TLRs increased significantly, indicating that the immune system of germ-free zebrafish was activated. Zebrafish larvae can induce several pro-inflammatory genes through the TLR/MyD88 signaling pathway, thereby increasing the resistance of the larvae (47). In this study, we focused on genes relating to MAPK and NF-κB inflammation signaling pathway, as well as related cytokines TNF-α, IL-6 and IL-1β. Other studies have found that in germ-free zebrafish, Lactobacillus casei BL23 in could effectively promote the expression of pro-inflammatory factors such as TNF-α, IL-10 and IL-1β to fight the infection with Aeromonas veronii (48, 49). Thus, the anti-infective effect of *L. casei* BL23 was made possible by an enhanced immune response against pathogens in germ-free zebrafish. Consistently, our results suggest that, compared with prior administration of ΔluxS, supplementation with the wild-type strain could significantly increase the expression of IL-1β pro-inflammatory factor and activate MAPK and NF-κB signaling pathway. We speculate that the wild-type strain improved resistance to an ETEC infection by maintaining a high level of pro-inflammatory signaling pathways in germ-free zebrafish, which may be attributed to the stronger ability of the wild type to colonize the intestine compared with ΔluxS. Another study tested different adhesion assays, such as competitive inhibition, adhesion inhibition and displacement on the strain LGG with good adhesive properties and showed that LGG all exhibited significant antagonistic activity against Escherichia coli serotype O26:H11 (50). Therefore, another reason the WT may be more resistant to ETEC infection might be that WT is a better competitor of EHEC compared to ΔluxS. It is worth mentioning that Escherichia coli (E. coli) as a Gram-negative bacterium can also participate in information exchange through AI-2 signaling molecules. The chemotaxis of AI-2 can mediate the collective behavior, for example autoaggregation, in *E.coli*, which enhances stress resistance and promotes the formation of biofilms (51, 52). Compared with the avian pathogenic Escherichia coli DE17 wild-type strain, the △luxS strain showed a reduced survival rate and virulence *in vivo* (53). We inferred that the AI-2/LuxS QS system not only enhanced the biofilm formation of LGG and immune regulation but may also influence the expression of virulent ETEC genes (Fig. 7). Unfortunately, the mechanism how LGG and ETEC communicate with each other, remains unclear. Thus, the communication between bacteria is a complex process due to their complicated microbial ecology.

In conclusion, AI-2 decreased sharply after deletion of the *luxS* gene in LGG, thus impairing adhesion, EPS synthesis, and biofilm formation. The effect may be related to the weaker anti-inflammatory response to LGG in germ-free zebrafish (Fig. 7). However, the communication between the host and the bacteria is a complicated process. How exactly AI-2 transfers information between different species and affects the immune system of organisms remains to be fully explored.

## MATERIALS AND METHODS

### Bacterial strains and growth conditions.

Lactobacillus rhamnosus GG (LGG; ATCC 53103) ΔluxS mutant were a gift from J.P. van Pijkeren’ lab (University of Wisconsin-Madison, Madison, USA). The method for constructing this mutant is provided in the supplementary information. In short, the upstream and downstream flanks of *luxS* were cloned into the vancomycin-based counterselection system (pVPL3002) by Ligase Cycling Reaction (LCR) (54). Lactobacillus rhamnosus ATCC 53103 ΔluxS was obtained by deleting *luxS* as described by Zhang et al. (55). The specific base sequence of the deleted *luxS* gene is shown in Fig. S2. Lactobacillus rhamnosus GG (LGG; ATCC 53103) and its ΔluxS strain were cultivated at 37°C in de Mann-Rogosa-Sharpe (MRS) broth under static conditions, and counted at different times after inoculation (56). Both strains used for detection of AI-2 were grown in modified MRS medium in which glucose was replaced by 1% galactose (57). Vibrio harveyi BB170 (ATCC BAA-1117) was cultivated at 37°C under shaking in autoinducer bioassay (AB) medium which was prepared as described previously (58). Enterotoxigenic Escherichia coli (ETEC) were cultured in LB medium under shaking at 37°C.

### Determination of stress resistance.

Characteristics of the strain such as hydrophobicity, auto-aggregation, and stress resistance to acid, bile salt, Zn^2+^ and Cu^2+^ were determined. The cells collected at the mid-exponential growth phase were washed with PBS, and the OD_600_ of the bacterial suspension was adjusted to 0.8 (A_o_). The bacterial solution was stored at room temperature, and the supernatant OD_600_ (A) was measured at different time points. The calculation formula for auto-aggregation is AA% = (A_0_ − A)/A × 100% (59). An equal volume of bacteria suspension was mixed with xylene for 2 min, and then allowed to rest for 30 min to determine the OD_600_ of the water phase. The hydrophobicity calculation formula is H% = [(H_o_ − H)/H_o_] × 100%, where H_o_ and H are the absorbance before and after xylene extraction (60). The activated *Lactobacilli* were cultured in MRS medium containing hydrochloric acid (pH = 3) and bile salt (0.3%) for 2 h and cultured in copper ion and zinc ion (100 mg/L) for 24 h, and their survival rate was determined by the plate counting method.

### High-throughput sequence analysis.

RNA was extracted from zebrafishes at 7 dpf according to a previous method (61). Each group consisted of 30 zebrafishes. All results were obtained from at least three replicates. The exact amount of 3 μg RNA per sample was used as input material. NanoDrop ND-1000 (NanoDrop, USA) was used to determine RNA quantity and purity. The RNA integrity was assessed by Bioanalyzer 2100 (Agilent, USA) with RIN number >7.0, and was confirmed by electrophoresis with a denaturing agarose gel. Library construction and RNA-seq were performed by LC-Bio Technology Co., Ltd., China. In addition, we performed 2 × 150 bp paired-end sequencing on an Illumina Novaseq 6000 following the vendor's recommended protocol. Raw data in fastq format were processed through in-house perl scripts. Both the building index of the reference genome and aligning the clean reads to the reference genome were carried out using Bowtie2-2.2.3 soft (62). Gene expression levels were quantified with HTSeq v0.6.1 by counting the numbers of the reads mapped to the corresponding gene. Differential expression analysis was performed by using the DESeq R package (1.18.0). The resulting *P* values were adjusted using Benjamini and Hochberg’s approach for controlling the false discovery rate. Genes with an adjusted *P* value <0.05 found by DESeq were considered as differentially expressed. We used KOBAS software to test the statistical enrichment of differentially expressed genes in the KEGG pathway. All sequences in this study were deposited in the NCBI sequence archive under the project number PRJNA796827.

### AI-2 bioassay.

The AI-2 bioassay was performed as described previously with some modifications (58). The test strains were centrifuged at 12000 g for 10 min at 4°C and the cell-free supernatant was collected. The pH of the supernatant was adjusted to 6.5 with 2M NaOH and filtered through a 0.22-μm filter (Millipore, USA). The presence of AI-2 in the preconditioned media was assayed using the V. harveyi BB170 (luxN::Tn5) reporter strain, which only responds to AI-2. The V. harveyi BB170 reporter strain was grown for 16 h in AB medium at 30°C with aeration (200 rpm), then washed and re-suspended in fresh AB medium to achieve OD600 of 0.5. The cell-free supernatant was mixed with a V. harveyi BB170 culture diluted 1000-fold at a ratio of 10% (vol/vol), and the mixture was added to a white, flat-bottomed 96-well microtiter plate (Corning, USA), followed by shaking in a rotary shaker at 200 rpm, at 30°C. Luminescence was measured every hour for 6 h in a fluorescent microtiter plate reader (Synergy HTX, BioTeK, USA).

### HPLC-FLD and LC-MS/MS.

4,5-dihydroxy 2,3-pentanedione (DPD; Omm Scientific Inc, USA) was diluted to concentrations in the range of 1–1000 ng/mL as standards. 2,3-Diaminonaphthalene (DAN; Aladdin Inc, China) was prepared by dissolving 10 mg DAN in 50 mL 0.1 M HCl. Standards or LGG culture supernatant was thoroughly mixed with an equal volume of DAN solution in a 2 mL autosampler vial for 2 min. Then, these samples were incubated in a water bath at 90°C for 40 min. After cooling, a 10 μL sample was injected into a HPLC system equipped with a fluorescence detector (Agilent 1100, USA). Separation was performed on a reverse-phase column (SunFire C18, Waters, Ireland) with column temperature at set 30°C. The mobile phase contained 0.1% formic acid (solvent A) and acetonitrile (solvent B) at a flow rate of 1 mL/min. The gradient elution curve was used as follows: *t* = 0 min, 95% solvent A, 5% solvent B; *t* = 5 min, 95% solvent A, 5% solvent B; and *t* = 40 min, 20% solvent A, 80% solvent B. The excitation and emission wave lengths of the fluorescence detector were set at 271 and 503 nm, respectively.

In parallel, the processed samples were analyzed by LC-MS/MS. Chromatographic separation was achieved on an ACQUITY UPLC HSS T3 column (1.8 μm, 2.1 × 150 mm; Waters Corp.) at a column temperature of 50°C by using a mobile phase of 0.1% formic acid-water and acetonitrile at a flow rate of 0.3 mL/min. The sample injection volume was 5 μL. The mass spectrometer used was an AB TripleTOF 5600plus System (AB SCIEX, Framingham, USA). The optimal MS conditions were as follow: Positive ion mode, source voltage was +5.5 kV, and the source temperature was 600°C. Declustering potential (DP), 100 V; collision energy (CE), 10 V. For MS/MS acquisition mode, the parameters were almost the same except that the collision energy (CE) was set at 40 ± 20 V, ion release delay (IRD) at 67, ion release width (IRW) at 25. The IDA-based auto-MS2 was performed on the 8 most intense metabolite ions in a full scan cycle (1 s). The scan range of *m/z* of the precursor ion and product ion was set as 100–1500 Da and 50–1500 Da.

### Bacterial adhesion assay.

Adhesion of wild-type and mutant LGG to porcine small intestinal epithelial cells (IPEC-J2) was examined as described previously (63). IPEC-J2 cells were cultured to confluence in an incubator at 37°C with 5% CO_2_ in DMEM/F12 medium containing 10% (vol/vol) fetal bovine serum in a 6-well plate. After the wells were washed three times with PBS, the same volume of LGG [5 × 10^8^ colony forming unites (CFU/mL)] and DMEM/F12 medium were added and cultured for 2 h. Then, the wells were washed three times with PBS to remove non-adherent bacteria. The adherent bacteria were fixed by 4% paraformaldehyde for 15 min and subjected to Gram staining. The adhesive bacteria were counted under a microscope.

### Determination of polysaccharides in biofilm matrix.

Polysaccharides in the biofilm matrix were quantified according to a previous study (64). Overnight broth cultures of LGG were diluted 1:100 with fresh sterile MRS medium, and the dilution was transferred into individual wells of 6-well plates at 37°C and cultured for 24 h. DPD (1 μM) as AI-2 precursor was added to the culture. The medium was removed, and the wells were washed gently with 0.85% physiological saline. The biofilms in the wells were re-dissolved in physiological saline and treated with a sonicator at 50 kHz for 5 min. The suspension was treated at 80°C for 30 min and centrifuged at 8000 × *g* for 15 min. The supernatant was then filtered through a 0.22-μm filter for the extraction of polysaccharides. The absorbance at 490 nm was measured, and polysaccharides (expressed as mg/mL) in the filtrate were quantified by using glucose as standard.

### Biofilm formation assay.

Biofilm formation was measured quantitatively by the crystal violet assay (65). Briefly, LGG was cultured for 16 h and diluted 1000-fold with fresh MRS medium. Samples were added at 1% (vol/vol) to the wells of a sterile 96-well flat-bottomed plastic culture plate with MRS medium. DPD (1 μM) as AI-2 precursor was added to the culture of either ΔluxS or wild-type strains. The plates were cultured anaerobically at 37°C for 24 h. The wells with broth medium only were used as negative control. The medium was discarded and the plates were washed three times with 0.85% physiological saline to remove loosely adherent cells. Biofilm was fixed with 200 μL of methanol for 10 min. The plates were stained with 0.2% (vol/vol) crystal violet for 15 min and washed three times with 0.85% physiological saline to remove unbound crystal violet dye. The dye bound to the biofilm was re-dissolved with 95% ethanol and the optical density at 595 nm was determined with a microplate reader (Synergy^H1^, BioTeK, USA).

### Confocal laser scanning microscopy (CLSM).

LGG was inoculated into a 6-well culture plate containing sterile glass slides and incubated anaerobically at 37°C for 24 h. DPD (1 μM) as AI-2 precursor was added to each culture. The slides were then removed and washed three times with sterile PBS. Prior to image acquisition, each biofilm was fluorescently labeled with 0.3% SYTO-9 and 0.3% PI (Sigma-Aldrich, China) in the dark for 15 min, then stained with calcofluor white (250 μg/mL, Sigma-Aldrich, China) for 5 min. These samples were observed with a confocal laser scanning microscope with an oil immersion 63 × objective lens (Carl Zeiss LSM880, Jena, Germany). Zeiss confocal software was used to analyze biofilm images, allowing for collection of z-stacks. Biofilm thickness and EPS bio-volume were calculated using COMSTAT (66).

### Generation of germ-free embryos and exposure to probiotic.

Zebrafish embryos were kindly provided by Professor JR Peng (Zhejiang University, China). Naturally bred eggs were collected immediately after being laid and soaked in a 0.1% PVP-I disinfectant solution for 1 min. The fish eggs were washed three times with a sterile culture solution. Then the fish eggs were soaked in a 0.3% calcium hypochlorite disinfectant solution for 10 min, and were washed 3 times with a sterile culture solution. Finally, they were transferred to a sterile dish with sterilized egg water containing 5 μg/mL kanamycin, 100 μg/mL ampicillin, and 250 ng/mL amphotericin B. Unfertilized embryos were removed on time during this stage. At day 4 post fertilization (dpf), the eggs were washed three times with sterile culture solution and incubated in it. Zebrafish larvae in germ-free exposed to none or to wild-type or ΔluxS LGG at a concentration of 10^8^ CFU/mL for 24 h on 5 dpf were designated as the GF, WT and ΔluxS group, respectively. These zebrafish larvae treated differently were further exposed to ETEC at a concentration of 10^8^ CFU/mL for 24 h on 6 dpf and considered as the ETEC, WT+ETEC and ΔluxS+ETEC groups, respectively.

### Counting of bacteria in zebrafish larvae.

The zebrafish larvae were treated with tricaine (4 mg/mL) and washed with sterile PBS for 3 times to remove bacteria loosely attached to the skin. They were transferred to tubes containing sterile glass beads (500 μm) and 500 μL PBS, and homogenized at 60 Hz for 60 sec. The suspension was serially diluted and incubated on the MRS plate at 37°C for 48 h. The results were presented as mean log_10_ CFU ± standard error of the mean (SEM) per five fish.

### Zebrafish imaging.

According to the manufacturer's instructions, 5-Carboxyfluorescein diacetate (5-CFDA, MCE, USA) stock solution (1 mM) with DMSO was prepared and stored at −20°C. LGG (10^8^ CFU/mL) were mixed with 5-CFDA to obtain the final concentration of 20 μM. The mix was incubated at 37°C for 15 min in the dark. The bacterial suspension was centrifuged at 8000 g at 4°C for 10 min, and the precipitated bacteria were washed with PBS for three times to remove unlabeled 5-CFDA. Zebrafish larvae (5 dpf) were co-incubated with fluorescently labeled LGG for 24 h, then incubated in sterilized water for 1 day. The colonization of LGG in larvae was observed with a fluorescence stereo microscope (Nikon SMZ18, Japan) on 7, 8 and 9 dpf, and the fluorescence intensity was analyzed with ImageJ (Rawak Software Inc., Stuttgart, Germany).

Samples consisting of 10 zebrafish larvae on 7 dpf were fixed with 2.5% glutaraldehyde in phosphate buffer (0.1 M, pH 7.0) for 4 h, washed in the same buffer at room temperature, and post fixed for 1 h in 1% osmium tetroxide. After the samples were dehydrated with a series of gradient ethanol, they were embedded in the Spurr resin mixture overnight. The samples were transferred to absolute acetone for 20 min and embedded in Spurr resin mixture for 12 h The specimens were sliced in a LEICA EM UC7 ultratome and sections were stained by uranyl acetate and alkaline lead citrate for 10 min respectively and observed with a transmission electron microscope (TECNAI T10, the Netherlands).

### Real-time PCR.

Total RNA was isolated from 6 replicates, each from 30 zebrafish larvae (7 dpf) with TRIzol reagent following manufacturer's instructions. High-throughput sequence analysis of zebrafish was conducted using the same method as described in 2.2. Quantitative PCR was performed with SYBR green Supermix (TaKaRa, Japan) on a CFX96 Real-Time PCR thermocycler (Bio-Rad). The reaction mixtures were incubated for 5 min at 95°C, followed by 40 cycles of 5 s at 95°C, 20 s at 60°C, and 30 s at 60°C. The melting curve was performed from 65°C to 95°C with a 0.5°C increment for 10 s. β-actin was used as the housekeeping gene. Data were analyzed by using the ΔΔCt method. The primers are listed in Table S4. All PCRs were performed with three biological replicates and each biological replicate included three technical replicates.

### Statistical analysis.

All data were combined and analyzed by one way ANOVA with a general linear model, followed by Duncan’s multiple range tests using Statistical Analysis System (SAS Institute, Cary, NC, USA). Significant differences were indicated at *P < *0.05 (*), *P < *0.01 (**) and *P < *0.001 (***). Different letters indicate significant differences within each strain (*P < *0.05). Data are presented as means ± SEM. Data were combined from at least three independent experiments.

### Data availability.

All transcriptome sequences of zebrafish with different treatment in this study were deposited in the NCBI sequence archive under the project number PRJNA796827.
