# Supplementary material for: AI-2/LuxS Quorum Sensing System Promotes Biofilm Formation of Lactobacillus rhamnosus GG and Enhances the Resistance to Enterotoxigenic Escherichia coli in Germ-Free Zebrafish
Source: Microbiol Spectr. 2022 Jun 14;10(4):e00610-22. doi: 10.1128/spectrum.00610-22 (PMC9430243; doi:10.1128/spectrum.00610-22)
Supplement: Supplemental file 1 — Supplemental material. Download spectrum.00610-22-s0001.pdf, PDF file, 0.5 MB [file spectrum.00610-22-s0001.pdf]

Supplementary Material and Methods

Construction of *L. rhamnosus* ATCC 53103 Δ*luxS*

To delete *luxS*, we applied the vancomycin-based counterselection system (pVPL3002) as described by Zhang *et al.* [1]. First, we cloned the upstream and downstream flanks of *luxS* in pVPL3002 by Ligase Cycling Reaction (LCR)[2]. The plasmid backbone of pVPL3002 was amplified with oVPL187-188, and oligonucleotide pairs oVPL3228-3229 and oVPL3230-3231 were used to amplify the up- and downstream flanks of *luxS,* respectively. The three amplicons were fused using bridging oligonucleotides oVPL3232, 3233, and 3234. The resulting plasmid construct was named pVPL31157. We transformed 3 μg pVPL31157 in *L. rhamnosus* ATCC 53103 as described in Zhang *et al.* We used oligonucleotide pairs oVPL49-3235-3236 and oVPL97-3235-3236 to identify upstream and downstream integration of pVPL31157, respectively. Upon confirmation of single-crossover homologous recombination (SCO), cells were cultured in MRS for 20 generations in the absence of antibiotics and plated on MRS agar containing 1,000 μg/mL vancomycin. This selects for cells that have undergone a second homologous recombination event. By using PCR (oligonucleotides oVPL3235-3236) we screened for deletion of *luxS*. Efficiency of the deletion was subsequently verified by Sanger sequencing. The resultant *luxS* mutant strain was named VPL4310. The strains, plasmids, and oligonucleotides used for *luxS* mutant construction are detailed in Table 1 and 2.

Supplementary Table S1. Bacterial strains and plasmids used in this study

**Genus and Species**	**Strain**[#]	**Description**[‡]	**Source**[+]
Escherichia coli	EC1000	In trans RepA provider, Kan^R (cloning host)	[3]
Escherichia coli	VPL3002	EC1000 harboring pVPL3002, Em^R	[1]
Escherichia coli	VPL31157	EC1000 harboring pVPL31157, Em^R	This study
Lactobacillus rhamnosus	ATCC 53103	Wild-type	ATCC
Lactobacillus rhamnosus	VPL4310	ATCC 53103 *ΔluxS*	This study
**Plasmids**	**Genotype**	**Description**	**Source**
pVPL3002	pORI19::*ddlA* $F258Y_{reuteri}$, Em^R	Suicide shuttle vector with vancomycin counter-selection marker	[1]
pVPL31157	pVPL3002::*luxS* deletion cassette, Em^R	Deletion cassette targets *luxS* in ATCC 53103	This study

[#]: VPLxxxx: Van Pijkeren Laboratory strain collection identification number; [‡]: Kan^R: kanamycin resistance; Em^R: erythromycin resistance; pVPLxxxx: Van Pijkeren Lab plasmid collection identification number; [+]: ATCC: American Type Culture Collection

Supplementary Table S2. Oligonucleotides used in this study

**Oligonucleotides**#	**Sequence (5'-3')**	**Description**‡
oVPL49	acaatttcacacaggaaacagc	Oligo paired with oVPL97 used for screening pVPL3002 constructs
oVPL97	cccccattaagtgccgagtgc	Oligo paired with oVPL49 used for screening pVPL3002 constructs
oVPL187	taccgagctcgaattcactgg	Rev, internal oligo for pVPL3002 backbone amplification
oVPL188	atcctctagagtcgacctgc	Fwd, internal oligo for pVPL3002 backbone amplification
oVPL3228	ttagctgatgtggtgcaagc	Fwd, paired with oVPL3229 used for *luxS* gene deletion cassette (u/s)
oVPL3229	taaggcgccttaactgcagtgtg	Rev, paired with oVPL3228 used for *luxS* gene deletion cassette (u/s)
oVPL3230	atttacccggcaggttgtctaatc	Fwd, paired with oVPL3231 used for *luxS* gene deletion cassette (d/s)
oVPL3231	gttcgtttatccgctgctgt	Rev, paired with oVPL3230 used for *luxS* gene deletion cassette (d/s)
oVPL3232	aaacgacggccagtgaattcgagctcggtattagct gatgtggtgcaagcatacgtggca	Bridging oligonucleotides used for LCR
oVPL3233	gttagatcacactgcagttaaggcgccttaatttaccc ggcaggttgtctaatctgacga	Bridging oligonucleotides used for LCR
oVPL3234	cggatttgaaacagcagcggataaacgaacatcctc tagagtcgacctgcaggcatgcaa	Bridging oligonucleotides used for LCR
oVPL3235	ggcgttactggcacctagac	Fwd, screening oligo paired with oVPL3236 used for screening *luxS* deletion
oVPL3236	ggttgagcatcggtcgtaat	Rev, screening oligo paired with oVPL3235 used for screening *luxS* deletion

#: oVPLxxxx: Van Pijkeren Laboratory oligonucleotide identification number; ‡: fwd: forward; rev: reverse; u/s: upstream; d/s: downstream; LCR: ligation cycling reaction

Supplementary data

Supplementary Table S3. Primer sequences for RT-qPCR

Primers	5'-3'	Sequence
**β-actin**	Forward	GTGCCCATCTATGAGGGTTACGCT
	Reverse	GTCACGGACAATTTCTCTTTCGGC
**TLR1**	Forward	TAAACCTTCGGCACAACCGA
	Reverse	AGATCCAGCAAGCGGTTGAA
**TLR2**	Forward	AAACCTGCTGTCGGTCGATT
	Reverse	ACACAGGGAAAACGAAGGCT
**TLR3**	Forward	TTTCTGGCCTTCGGAACCTG
	Reverse	ACTTGTTGATGCCCATGCCT
**TLR4b**	Forward	TCACACCGTTGTGCTTCGAG
	Reverse	AATGTGGATCGGATGTCCAGC
**TLR5b**	Forward	AGAGACGGGCGCGTTTAAT
	Reverse	GAAGCTGGCTGGATTTTCTGT
**TNF-α**	Forward	GTGCAATCCGCTCAATCTGCACG
	Reverse	AATGGAAGGCAGCGCCGAGG
**IL-1β**	Forward	GTCCACGTATGCGTCGCCCA
	Reverse	GGGGCAACAGGCCAGGTACA
**IL-6**	Forward	ATGACGGCATTTGAAGGG
	Reverse	GCAGCGGTCTGAAGGTTT
**NF-kB**	Forward	GAGCCCTTTGTGCAAGAGAC
	Reverse	TGGGATACGTCCTCCTGTTC
**IκBα**	Forward	TTTCGGAGGAGATGGAGAGA
	Reverse	CTGTTCAGGTACGGGTCGTT
**MyD88**	Forward	GAGGATGGTGGTGGTCATCT
	Reverse	CGACAGGGATTAGCCGTTTA
**STAT**	Forward	ATCGACCTTGAGACGCACTC
	Reverse	CCCATGCGTTTGGCATTTGA
**JNK**	Forward	GGGAATAGTGTGTGCTGGATATGATG
	Reverse	TGGTTCTGGAAGGGTCTGCTGAG
**p38**	Forward	CCTGAGATCATGCTCAACTGG
	Reverse	GCTAGGCATCCTGCTTATTAGAGAG

References

1. Zhang, S., et al., *D-Ala-D-Ala ligase as a broad host-range counterselection marker in vancomycin-resistant lactic acid bacteria.* Journal of bacteriology, 2018: p. JB. 00607-17.
2. Kok, S.d., et al., *Rapid and reliable DNA assembly via ligase cycling reaction.* ACS synthetic biology, 2014. **3**(2): p. 97-106.
3. Leenhouts, K., et al., *Construction of a food-grade multiple-copy integration system for Lactococcus lactis.* Applied Microbiology and Biotechnology, 1998. **49**(4): p. 417-423.

Supplementary Figure S1. Expression of TLRs of germ-free (GF), conventionally raised (CR), and LGG-colonized zebrafish larvae, n=6. GF, WT and ΔluxS, zebrafish larvae in germ-free were exposed to none or to wild-type or ΔluxS LGG at a concentration of 10^8 cfu/mL for 24 h on 5 dpf, respectively. TLR, toll-like receptor, $p < 0.05$ (*), $p < 0.01$ (**) and $p < 0.001$ (***).

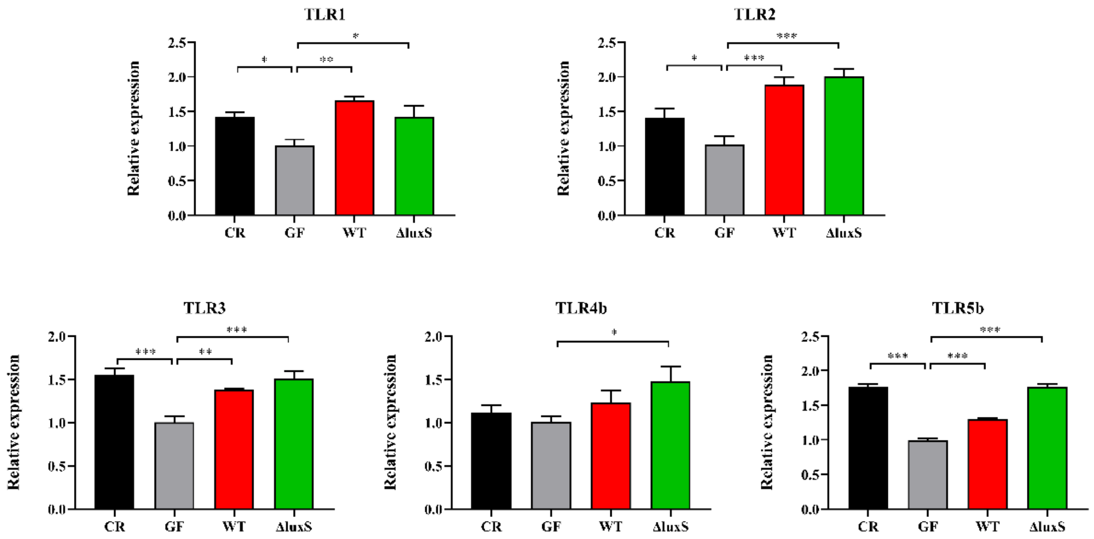

Supplementary Figure S2. The deletion base of the *luxS* gene.

```
WT.txt      AGTTAAGGCGCCTTATGTCCGGTTAATTACCACCGAAAAAGGCCCGAATGGCGATGAAATTTCTAATTATGATTTACGCT
luxS.txt    AGTTAAGGCGCCTTA.................................................................
Consensus   agttaaggcgcctta

WT.txt      TGGTTCAGCCAAACACCGCTGCGATTGATACTGCCGGGTTGCATACAATTGAACACTTGTTGGCATCATTATTGCGCGAT
luxS.txt    ................................................................................
Consensus

WT.txt      CGCATGGATGGCGTGATTGATTGTTCACCGTTTGGCTGCCGCACCGGCTTTCACTTGATTACCTGGGGAACACACAGCAC
luxS.txt    ................................................................................
Consensus

WT.txt      CACGGAAGTCGCCAAAGCGCTGAAGTCCAGCCTTGAAGCGATTGCTAACGATATTACCTGGGATGATGTACCCGGTGTTG
luxS.txt    ................................................................................
Consensus

WT.txt      ACATTAAGAGTTGTGGCAACTACAAAGATCACAGCCTGTTTTCTGCTAAAGAGTGGGCAAAGTTGATTTTGAGTCGCGGC
luxS.txt    ................................................................................
Consensus

WT.txt      ATTTCTAACGATCCATTTACCCGGCAGGTTGTCTAATCTGACGAGCATTGATTAGAACCAAAAATGGCACTGCACAAGTT
luxS.txt    ..............ATTTACCCGGCAGGTTGTCTAATCTGACGAGCATTGATTAGAACCAAAAATGGCACTGCACAAGTT
Consensus                 atttacccggcaggttgtctaatctgacgagcattgattagaaccaaaaatggcactgcacaagtt
```
